# Substrate stiffness regulates the differentiation profile and functions of osteoclasts via cytoskeletal arrangement

**DOI:** 10.1111/cpr.13172

**Published:** 2021-12-24

**Authors:** Qingxuan Wang, Jing Xie, Chenchen Zhou, Wenli Lai

**Affiliations:** ^1^ State Key Laboratory of Oral Diseases West China Hospital of Stomatology Sichuan University Chengdu China; ^2^ Department of Orthodontics West China Hospital of Stomatology Sichuan University Chengdu China; ^3^ Department of Pediatric Dentistry West China Hospital of Stomatology Sichuan University Chengdu China

**Keywords:** cytoskeletons, osteoclast differentiation, osteoclasts, PDMS substrates, substrate stiffness

## Abstract

**Objectives:**

Aging and common diseases alter the stiffness of bone tissue, causing changes to the microenvironment of the mechanosensitive bone cells. Osteoclasts, the sole bone‐resorbing cells, play a vital role in bone remodeling. This study was performed to elucidate the mechanism through which osteoclasts sense and react to substrate stiffness signals.

**Materials and methods:**

We fabricated polydimethylsiloxane (PDMS) substrates of different stiffness degrees for osteoclast formation progressed from osteoclast precursors including bone marrow‐derived macrophages (BMMs) and RAW264.7 monocytes. Osteoclast differentiation in response to the stiffness signals was determined by examining the cell morphology, fusion/fission activities, transcriptional profile, and resorption function. Cytoskeletal changes and mechanosensitive adhesion molecules were also assessed.

**Results:**

Stiffer PDMS substrates accelerated osteoclast differentiation, firstly observed by variations in their morphology and fusion/fission activities. Upregulation of canonical osteoclast markers (Nfatc1, Acp5, Ctsk, Camk2a, Mmp9, Rela, and Traf6) and the fusion master regulator DC‐stamp were detected on stiffer substrates, with similar increases in their bone resorption functions. Additionally, the activation of cytoskeleton‐associated adhesion molecules, including fibronectin and integrin αvβ3, followed by biochemical signaling cascades of paxillin, FAK, PKC, and RhoA, was detected on the stiffer substrates.

**Conclusions:**

This is the first study to provide evidence proving that extracellular substrate stiffness is a strong determinant of osteoclast differentiation and functions. Higher stiffness upregulated the differentiation profile and activity of osteoclasts, revealing the mechanical regulation of osteoclast activity in bone homeostasis and diseases.

## Graphical abstract

We fabricated a mechanical model of PDMS substrates that mimic physiologically mechanical properties of extracellular microenvironment, with varied stiffness for cell culture. Osteoclasts respond to stiffness variations in cell morphology, fusion/fission activity, osteoclast differentiation profile (Nfatc1, Acp5, Ctsk, Camk2a, Mmp9, Rela, Traf6, and DC‐stamp) and resorption functions. The potential regulatory mechanism with the involvement of cytoskeletons adhesion molecules: cytoskeletons adhesion molecules, including fibronectin‐integrin αvβ3 activation and following biochemical signaling cascades of paxillin, FAK, PKC, and RhoA were altered in response to stiffness signals. Bioinformatics proposed strong connection between cytoskeleton adhesion and osteoclast differentiation.

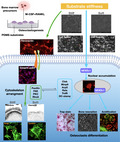

## 1 INTRODUCTION

Bones are in a constant state of dynamic development, undergoing sustained modeling and remodeling. Mechanical force is an important factor for stimulating bone metabolism and regulating bone structure and mass. A steady balance between new osteoid deposition by osteoblasts and bone resorption by osteoclasts is essential for the maintenance of skeletal homeostasis.^1^ The continuous reconstruction of normal bone tissue, development of new bone, and repair of traumatic bone defects are strongly regulated by physiological stimulation and external mechanical forces.^2^ Bone cells are mechanosensitive, under too little stress (eg, disuse of arms and space travel), a reduction in bone mass and the development of osteoporosis will occur, whereas under excessive mechanical force, bone hyperplasia, sclerosis, and abnormal woven bone structures can ensue. It is only under moderate mechanical stress (ie, within the physiological range) that effective bone homeostasis can be maintained and bone tissue growth and reconstruction promoted.^3^, ^4^

Osteoclasts are multinucleated giant cells derived from precursors of the monocyte/macrophage lineage. As the only bone‐resorbing cells in the human body, they play a vital role in maintaining bone metabolism, with osteoclastogenesis being the starting point in every round of the bone remodeling process.^5^ Osteoclast formation is regulated by two critical cytokines: macrophage colony‐stimulating factor (M‐CSF), which ensures the survival of osteoclast precursor cells; and receptor activator of nuclear factor‐kappa B (NF‐κB) ligand (RANKL), which drives the downstream signaling of transcription factors for osteoclastogenesis.^6^ Excessive osteoclast differentiation can lead to pathological bone loss, such as in age‐related osteoporosis, Paget's disease, and inflammatory rheumatic arthritis. Conversely, the restrained activity of these cells causes a significant increase in bone density.^7^ Therefore, understanding the activity of osteoclasts under multiple stimulation modes is a prerequisite to deciphering bone physiology and pathology. Alteration of the mechanical properties of bone tissue by aging and common diseases changes the mechanical microenvironment of the bone cells.^8^ One important mechanical signal from bone cell surroundings, the extracellular matrix (ECM) stiffness, was confirmed in a landmark study to be a strong determinant of the fate of mesenchymal stem cell differentiation.^9^ The mechanosensitive nature of bone cells and the stimulating effects of matrix stiffness on osteoblasts, osteocytes, and chondrocytes have been extensively studied.^10^, ^11^, ^12^ Although osteoclast differentiation has been proven to be influenced by multiple mechanical stimuli, such as tension force,^13^ microgravity,^14^ fluid shear stress,^15^ vibration,^16^ and compressive forces,^17^ the activity of osteoclasts in response to different degrees of microenvironmental stiffness remains unclear.

In this study, we generated five PDMS substrates, each of a different stiffness degree, to mimic the physiological mechanical properties of the extracellular microenvironment to determine how osteoclasts sense and react to such stimuli. We provide evidence proving that extracellular substrate stiffness is a strong determinant of the morphology, fusion activity, transcription profile, and resorption functions of osteoclasts. The levels of mechanosensitive cytoskeleton‐associated adhesion molecules were altered in response to substrate stiffness, with possible connections to osteoclast differentiation. Our findings add to existing knowledge about osteoclast activities under physical forces and their participation in bone homeostasis.

## 2 MATERIALS AND METHODS

### 2.1 Fabrication and characterization of polydimethylsiloxane substrates

The rigidity of PDMS can be regulated by changing the mass ratio of the curing agent to the liquid oligomeric base (Sylgard 184, Corning). Five such mass ratios were applied: 1:5, 1:15, 1:30, 1:45, and 1:60. The substrates were processed according to a previously described method.^18^ Although the rigidity of PDMS substrates is defined by the modulus of elasticity, we use the terms stiffness and Young's modulus (*E*) interchangeably. Mechanical tensile tests were conducted on all substrates using a universal testing machine (5967, Instron). In the linear elastic stage, *E* is defined as the ratio of applied stress to resultant strain according to Hooke's law,^19^ *E *= σ/ε × σ, which is the force per area (F/S), where ε indicates the stain defined by the relative elongation (DL/L) resulting from the external force.

### 2.2 In vitro osteoclastogenesis

All the animal experiments were approved by the Ethics Committee of West China Hospital of Stomatology (WCHSIRB‐D‐2017‐029). C57BL/6 mice were dissected to acquire femurs and tibias. Then, the bone marrow cells were flushed into a culture dish and cultivated for 24 h in complete α‐MEM (HyClone) supplemented with 10% FBS and 1% penicillin–streptomycin (HyClone) at 37°C under 5% CO_2_. Then, the cells were cultured for 72 h in complete medium containing 30 ng/ml M‐CSF (Catalog#416‐ML, R&D Systems), whereupon they were regarded as bone marrow‐derived macrophages (BMMs). These macrophages and RAW 264.7 monocytic cells (Shanghai Cell Center) were subsequently seeded onto the PDMS substrates in dishes and cultured in complete α‐MEM supplemented with 30 ng/ml M‐CSF and 100 ng/ml RANKL (Catalog#462‐TEC, R&D Systems). After 7 days of culture, during which the media containing inducing factors were replaced three times, osteoclastogenesis assays were performed to identify TRAP‐positive multinucleated cells (nuclei number ≥3) using an acid phosphatase staining kit (387A, Sigma‐Aldrich).

### 2.3 Atomic force microscopy

Atomic force microscopy (AFM) (SPM9700, Shimadzu) was applied for the surface test as previously described.^20^

### 2.4 Scanning electron microscopy

For scanning electron microscopy (SEM) analysis, osteoclasts cultured on PDMS substrates were first fixed in 2.5% glutaraldehyde and then dehydrated with a graded series of ethyl alcohol (30%, 50%, 70%, 80%, 90%, and 100%). Then, the specimens and blank PDMS substrates were coated with gold and examined using a scanning electron microscope (HT770, Hitachi).

### 2.5 Transcriptome sequencing and bioinformatics analysis

Total RNA was extracted from osteoclasts cultured on stiff (1:5) and soft (1:45) PDMS substrates (with three independent repeats), using Trizol reagent (Catalog#15596026, Invitrogen), and the quality was examined with an RNA Nano 6000 assay kit (Bioanalyzer 2100 System, Agilent Technologies). The Illumina NeoPrep system was applied to purify and fragment the mRNAs, synthesize cDNAs, and amplify the targets. Sequencing was accomplished with the Illumina NovaSeq 6000 platform, and the raw data were mapped and annotated referring to GRCm38/mm10 mouse genome from UCSC website with TopHat 2.1.0. Gene reads were counted using featureCounts (v1.5.0‐p3) and normalized to FPKM values. DESeq2 in the R package (1.20.0) was applied to identify differentially expressed genes (DEGs) in the stiff (1:5) and soft (1:45) groups, with padj ≤0.05 set as the threshold. The DEGs were then functionally annotated using the Gene Ontology and KEGG databases.

### 2.6 Bone resorption assay and acridine orange staining

BMMs (5 × 10^4^ cells/well) were seeded onto bovine cortical bone slices (either untreated [stiff bone slices] or decalcified overnight using 5% EDTA) and cultured for 14 days. Then, the cells were removed with 0.25 M ammonium hydroxide, and the bone slices were observed by SEM (JSM‐7500F, JEOL). ImageJ software was used to measure the percentage of resorbed areas in three random sections. Acridine orange (AO) staining of the bone slices was performed as described previously.^21^ In brief, following osteoclast differentiation, the cells were stained with 1 μM AO (Catalog#HY‐101879, MedChemExpress) at 37°C for 20 min, rinsed with phosphate‐buffered saline, and finally imaged by confocal laser scanning microscopy (CLSM) (FV3000, Olympus).

### 2.7 Immunofluorescence and confocal laser scanning microscopy

Osteoclasts were fixed with 4% paraformaldehyde for 20 min and then blocked with 5% BSA for 1 h. The cells were incubated overnight at 4°C with primary antibodies against the following proteins: nuclear factor‐activated T cells c1 (NFATc1) (Catalog#MA3‐024, Invitrogen), cathepsin K (CTSK) (Catalog#ab188604, Abcam), dendritic cell‐specific transmembrane protein (DC‐STAMP) domain containing 1 (DCST1) (Catalog#orb2242, Biorbyt), integrin beta‐3 (ITGB3) (Catalog#Ab‐773, Sigma‐Aldrich), and fibronectin 1 (FN1) (Catalog#MA5‐11981, Invitrogen). On the next day, the samples were incubated with Alexa Fluor 647 donkey anti‐rabbit (Catalog#ab150075, Abcam) and Alexa Fluor 647 goat anti‐mouse (Catalog#ab150115, Abcam) IgG secondary antibodies at ambient temperature for 2 h. After staining the cell nuclei and cytoskeleton with DAPI (Catalog#aD9642, Sigma‐Aldrich) and FITC‐labeled phalloidin (Catalog#F432, Invitrogen), the samples were sealed with 50% glycerol. All immunofluorescence images were captured by CLSM (FV3000, Olympus).

### 2.8 Quantitative reverse transcription PCR

Total RNA was extracted from the osteoclasts using Trizol reagent and then purified with the RNeasyPlus Mini Kit (Qiagen). The extracted RNA samples were quantified and then reverse transcribed to cDNA using a reverse transcriptase kit (Takara). The quantitative real‐time polymerase chain reaction (qPCR) was then performed with the cDNA, SYBR Green (Takara), and primers targeting the following genes: tumor necrosis factor receptor‐associated factor 6 (*Traf6*) (forward‐GGAGTTTGACCCACCTCTGG, reverse‐TGTGCCCTGCATCCCTTATG), acid phosphatase 5 (*Acp5*) (forward‐CCCACCGCCAAGATGGATTC, reverse‐AGCCACAAATCTCAGGGTGG), calcium/calmodulin‐dependent protein kinase II alpha (*Camk2a*) (forward‐ACAGAGCCATCCCCGAGACT, reverse‐GGTGCTCTCAGAAGATTCCTTCAC), matrix metalloproteinase‐9 (*Mmp9*) (forward‐TGTCATCCAGTTTGGTGTCG, reverse‐AATGGGCATCTCCCTGAAC), and glyceraldehyde 3‐phosphate dehydrogenase (*Gapdh*) (forward‐GGGTCCCAGCTTAGGTTCATC, reverse‐AATCCGTTCACACCGACCTT). All primer sequences were determined using the BLAST program. The qPCR conditions were 95°C for 10 min, followed by 45 cycles of 95°C for 5 s and 60°C for 30 s. The relative change in gene expression level was quantified using the $2^{‐\Delta \Delta C_{t}}$ method.

### 2.9 Western blot assay

Osteoclast lysates were obtained using RIPA lysis buffer (Catalog#R0020, Solarbio) containing PMSF (Catalog#P7626, Sigma‐Aldrich). After quantifying the total protein with a BCA protein assay kit, the sample was mixed with loading buffer and DTT (Catalog#D1070, Solarbio) and boiled for 6 min at 100°C. The proteins were then separated using 8%–12% SDS‐PAGE and transferred to polyvinylidene fluoride membranes. After blocking with 5% BSA, the membranes were incubated overnight with primary antibodies against the following proteins: NFATc1, CTSK, DCST1, ITGB3, FN1, focal adhesion kinase (FAK) (Catalog#ab219363, Abcam), NF‐κB p65 (Catalog#ab19870, Abcam), Ras homolog family member A (RhoA) (Catalog#ab187027, Abcam), phosphoprotein kinase C (p‐PKC) (Catalog#Thr638, ZenBio), and β‐actin as the internal reference (Catalog#ab6276, Abcam). Thereafter, the secondary antibody was applied for 2 h and the protein bands were visualized using an enhanced chemiluminescence reagent. ImageJ software was used for evaluating the gray value of each band.

### 2.10 Protein–protein interaction network analysis

A protein–protein interaction network was built by importing 15 target DEGs into the STRING database (v11.5) for analysis (https://string‐db.org). The target genes were clustered into two groups using the k‐means method, and connections of high confidence (cutoff edge = 0.700) were shown.^22^

### 2.11 Statistical analysis

All data are presented as the mean ±standard deviation and representative of three independent experiments. The Student's *t*‐test was used to evaluate differences between groups, with a *p* value of less than 0.05 indicating statistical significance.

## 3 RESULTS

### 3.1 Surface topography and elastic stiffness of the polydimethylsiloxane substrates

**FIGURE 1:**
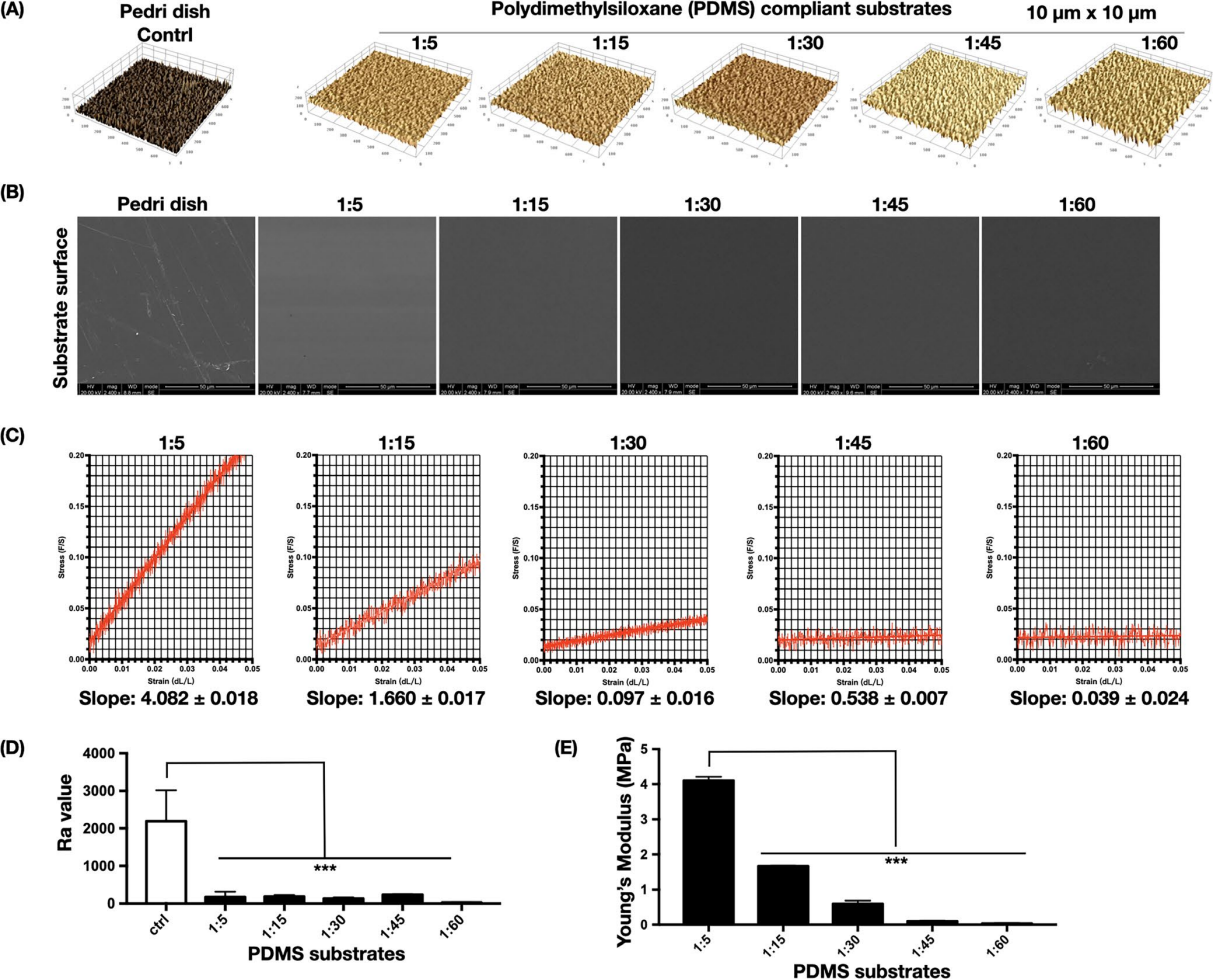
Basic characteristics of the PDMS substrates. (A) Representative surface topography of PDMS modified by different mixing ratios, in an area of 10 × 10 μm (*n* = 3 images). (B) SEM images of the morphology of dopamine‐coated PDMS substrates of different mixing ratios (*n* = 3 images). (C) Representative stress–strain curves of different PDMS substrates obtained through tensile testing. The scatter points (*n* = 500) are derived from the linear elastic region. The slope value is calculated by linear regression of the scatter points, with a 95% confidence interval. (D) PDMS surface *R*a parameter (*n* = 3 independent experiments). Significant difference compared with Petri dish. *p < .05, **p < .01, **p < .001. (E) Young's moduli of the PDMS substrates (*n* = 6 independent experiments). *Significant difference compared with 1:5 PDMS substrate. *p < .05, **p < .01, **p < .001

Five PDMS substrates, each of a different degree of stiffness, were prepared by increasing the ratio of curing agent to elastomer (1:5, 1:15, 1:30, 1:45, and 1:60). The substrate nanotopography, a key factor of biomaterials that influences cell behavior,^23^ was investigated by AFM (Figure 1A). The *R*a value, representing surface roughness, was lower for the substrates than for a Petri dish (Figure 1D). In the SEM images, the substrates had a relatively smooth surface morphology (Figure 1B), verifying the AFM results. The various substrates were subjected to mechanical tensile tests to measure their Young's modulus (*E*). Upon tension loading, the substrates exhibited a stress–strain response in sequence of the elastic stage, followed by the plastic stage that ended abruptly at a fracture strain.^24^ The linear region of the elastic stage of the stress–strain curve for each substrate is shown in Figure 1C. The slope was calculated from the linear regression line of the scatter points. The tensile elastic modulus of the five substrates decreased from 4.05 MPa to 1.66, 0.45, 0.10, and 0.03 MPa, respectively, in the order of stiffest to softest substrates (Figure 1E).

### 3.2 Osteoclasts displayed a distinct morphology and fusion activity on PDMS substrates of different stiffness degrees

**FIGURE 2:**
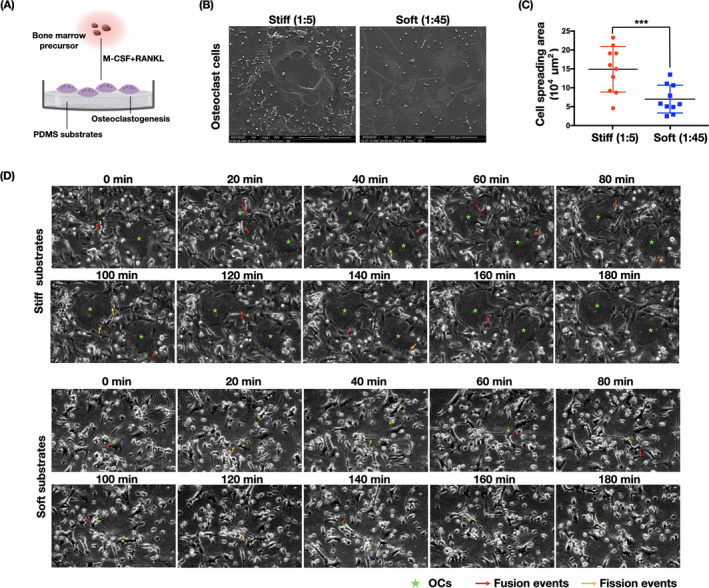
Changes in osteoclast morphology and fusion/fission activities under stiffness stimulation. (A) Schematic diagram illustrating bone marrow precursor cells undergoing osteogenic differentiation on PDMS substrates. (B) Morphologies of osteoclasts regulated by PDMS substrates of different stiffness degress (*n* = 3 independent SEM experiments). (C) Quantification of cell spreading regions in (B) (*n* = 10 independent quantification). Data are the mean ±SD. ***p < .001. (D) Bone marrow precursor cells were cultured on PDMS substrates of different rigidity (culture media containing M‐CSF and RANKL) for 7 days. Thereafter, live cell imaging was recorded for 24 continuous hours. Osteoclast fusion and fission events were observed. The image is sectioned from the subsequent 24‐h recorded series (*n* = 5 biological replicates). Time intervals of the individual images are noted above each image. Fusion events are indicated in red arrows and fission events in yellow arrows, and osteoclasts are marked with a green star

Bone marrow precursor cells were isolated, seeded onto the different PDMS substrates, and stimulated in osteoclast‐promoting medium containing RANKL and M‐CSF (Figure 2A). Once the cells had undergone osteoclast differentiation, SEM was used to detect cell morphology differences (Figure 2B). Substrates 1:5 (*E* = ~4.05 MPa) and 1:45 (*E* = ~0.1 MPa) were selected and termed as stiff and soft, which are in consistence with physiological nature of bone tissue.^9^ The stiffer substrate (1:5) had directed the formation of wide spread‐out osteoclasts, a sign of a more mature state. By contrast, cells cultured on a soft substrate (1:45) displayed a more prominently shrunken state and smaller size. The difference in cell spreading areas between the stiff and soft substrates was statistically significant (Figure 2C).

The differentiation of precursor cells into large osteoclasts is critical for bone resorption functions and is highly programmed by the fusion process.^25^ Bone marrow precursor cells were cultured for 7 days on stiff and soft substrates in the presence of M‐CSF and RANKL and then monitored by live cell imaging recording for 24 h. Fusion events were observed as two cells approaching each other, making close contact, and merging into one cell, whereas fission events were seen as the breaking apart of two tenuously connected cells into separate osteoclasts. On the stiff substrates, osteoclasts displayed a very flexible migration rate, with relatively small osteoclasts transforming into large osteoclasts through rapid fusion (~80 min) with surrounding cells (first row, Figure 2D). However, on soft substrates, although small osteoclasts formed long plasma protrusions with multiple cell contacts, they were unable to form large osteoclasts. Overall, osteoclasts cultured on the PDMS substrates changed dramatically in the fusion process, transforming to a new round of fission and returning to each other by rapid fusion, as shown in the full video (Movie S1 (stiff) and S2 (soft)).

### 3.3 Expression profile of osteoclast‐specific markers was enhanced on stiffer substrates

**FIGURE 3:**
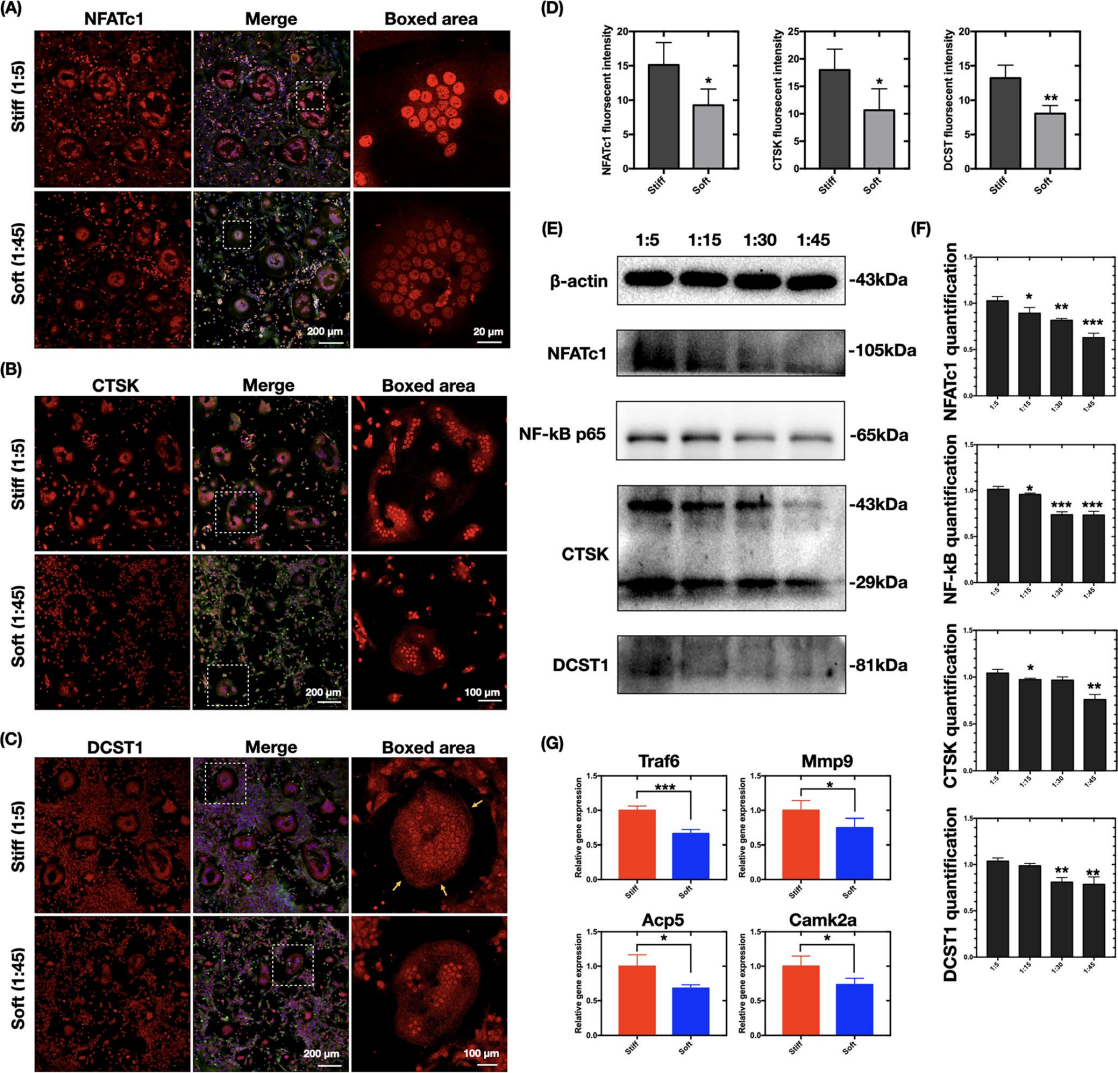
Substrate stiffness regulates the expression profile of osteoclast‐specific markers. (A–C) CLSM image of immunofluorescence‐stained cells (*n* = 3 independent experiments) showing changes in the expression levels of NFATc1, CTSK, and DCST1 (by red stain) on PDMS substrates of different rigidity. The osteoclasts were counterstained for F‐actin (phalloidin, green) and nuclei (DAPI, blue). (D) ImageJ quantification of the fluorescence intensity in (A–C) (*n* = 10 cells per group). Data are the mean ±SD. *p < .05, **p < .01. (E) Western blot assay of NFATc1, NF‐κB p65, CTSK, and DCST1 levels in cells cultured on PDMS substrates of different rigidity (*n* = 3 independent experiments). (F) Quantification of NFATc1, NF‐κB p65, CTSK, and DCST1 in (F). Data are the mean ±SD. Significant difference relative to group (1:5). *p < .05, **p < .01, **p < .001. (G) qPCR analysis of *Traf6*, *Mmp9*, *Acp5*, and *Camk2a* expression levels in stiff (1:5) and soft (1:45) groups (*n* = 3 independent experiments). *p < .05, ***p < .001

We used immunofluorescence staining to analyze osteoclast‐specific markers that control the pathways of cell fate during osteoclastogenesis. NFATc1, a master regulator of osteoclastogenesis, was significantly accumulated in the nuclear region (Figure 3A), as confirmed quantitatively by its total fluorescence intensity (Figure 3D) and western blot‐assayed level (Figure 3E). The expression levels of NF‐κB p65, which is important for the initial stimulation of NFATc1 in RANKL‐induced osteoclastogenesis,^6^ were higher on the stiffer substrates, as determined by western blotting (Figure 3E, 3F). Expression and distribution of CTSK and DCST1 was also explored (Figure 3B, 3C). DCST1 was more highly accumulated on the cell border of osteoclasts on the stiffer substrates. Western blotting showed the reduced expression of CTSK and DCST1 on the soft substrates relative to that on the stiff substrates (Figure 3E, 3F). Additionally, qPCR analysis of the osteoclast‐specific marker genes *Traf6*, *Mmp9*, *Acp5*, and *Camk2a* confirmed that the transcription levels were significantly higher on the stiffer substrates (Figure 3G). Collectively, these results suggest that stiffer substrates enhance osteoclast differentiation.

### 3.4 Stiffer substrates promoted the bone resorption function of osteoclasts

**FIGURE 4:**
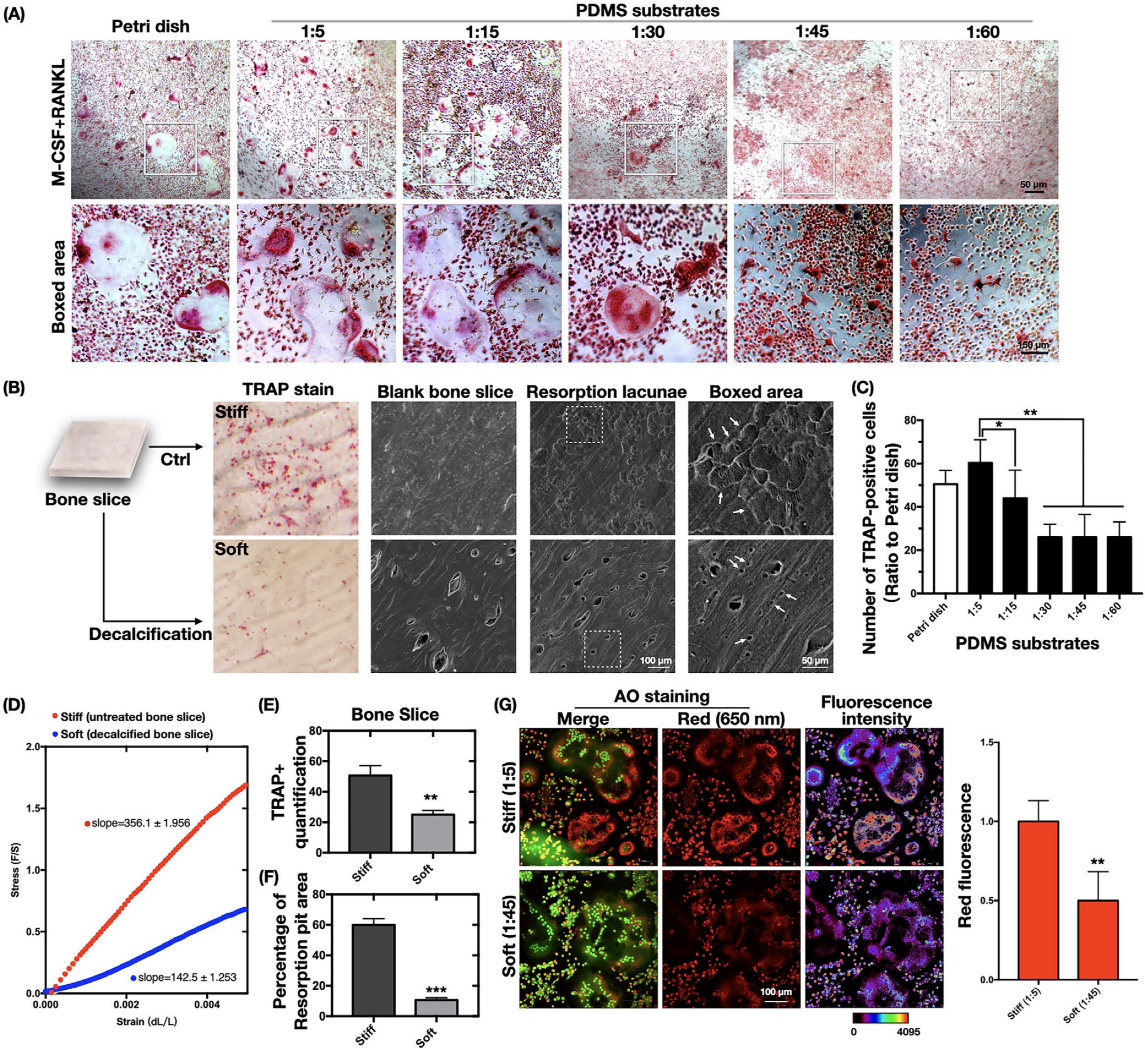
Substrate stiffness induces bone absorption function changes in osteoclasts. (A) Representative images of osteoclasts with TRAP staining after induction with M‐CSF and RANKL for 7 days (*n* = 3 independent experiments). (B) The same experiment in (A) was carried out with cells cultured on untreated bone slices (stiff) or decalcified bone slices (soft). The left column displays representative TRAP‐stained osteoclasts on the bone slices. The second column shows SEM images of untreated bone slices (stiff) and decalcified bone slices (soft) (*n* = 3 independent experiments). SEM images of bone resorption pits (indicated by white arrows) are displayed on the two right‐most columns. (C) Quantification of TRAP‐positive cells in (A). Data are the mean ±SD (*n* = 3). *p < .05, **p < .01. (D) Representative stress–strain curves of differently treated bone slices, obtained by tensile testing. The scatter points (*n* = 100) are derived from the linear elastic region. (E) Quantification of TRAP‐positive cells in (B). Data are the mean ±SD (*n* = 3). **p < .01. (F) ImageJ quantification of the resorption area measurements in (B). Data are the mean ±SD (*n* = 3). ***p < .001. (G) CLSM images of acridine orange (AO)‐stained (ie, acidified) osteoclasts cultured on PDMS substrates (*n* = 3 independent experiment). The red fluorescence of cytoplasmic vesicles indicates an acidic pH, whereas green‐stained nuclei indicate a neutral pH. The calibration bar indicates the false color correspondence to the 12‐bit pixel intensities of the right‐most columns. ImageJ was used to quantify the red fluorescence (*n* = 3). **p < .01

Next, we examined the bone resorption function of osteoclasts cultured on the different substrates. The number of multinucleated TRAP‐positive cells and the size of the cell spread were significantly decreased with the reduction in substrate stiffness (Figure 4A, 4C). To further confirm these results, the mechanical difference between untreated stiff bone slices and decalcified relatively softer bone slices was confirmed by tensile testing (Figure 4D). The slope value was almost identical to the Young's modulus of the bone slices, being significantly higher for the stiff sample (356.1 ± 1.956 MPa) than for the softer one (142.5 ± 1.253 MPa). The number of TRAP‐positive cells was also significantly higher on the stiff bone slices (Figure 4B, 4E). After cell removal, the bone resorption pit was imaged by SEM (Figure 4B, right‐most column). Of note, because decalcification caused fusiform notches on the surface of the soft bone slice (Figure 4B, middle column), absorption lacunae were clarified after the subtraction of these spots. The percentage of bone resorption areas was significantly higher in the stiff bone slice group (Figure 4F). The acidification of mature osteoclasts is a critical step for destroying extracellular bone matrixes.^7^ To further clarify the effects of substrate stiffness on the acidification of osteoclasts, staining with the acidic indicator AO was applied, whereupon acidic components in live cells appear as orange/red fluorescence while nuclei are stained green.^26^ Osteoclasts derived from the stiff substrates showed bright red fluorescence, indicating a higher level of cellular acidification (Figure 4G). The fluorescence intensity of OA was visualized as a 12‐bit pixel image in false color (Figure 4G, right‐most column), and differences were graphically quantified to be statistically significant. The results suggest that stiffer substrates stimulate terminal osteoclast differentiation by increasing bone resorption functions in cells.

### 3.5 Regulation of osteoclast differentiation by substrate stiffness may be related to integrin–extracellular matrix signaling pathways of cytoskeletal organization

**FIGURE 5:**
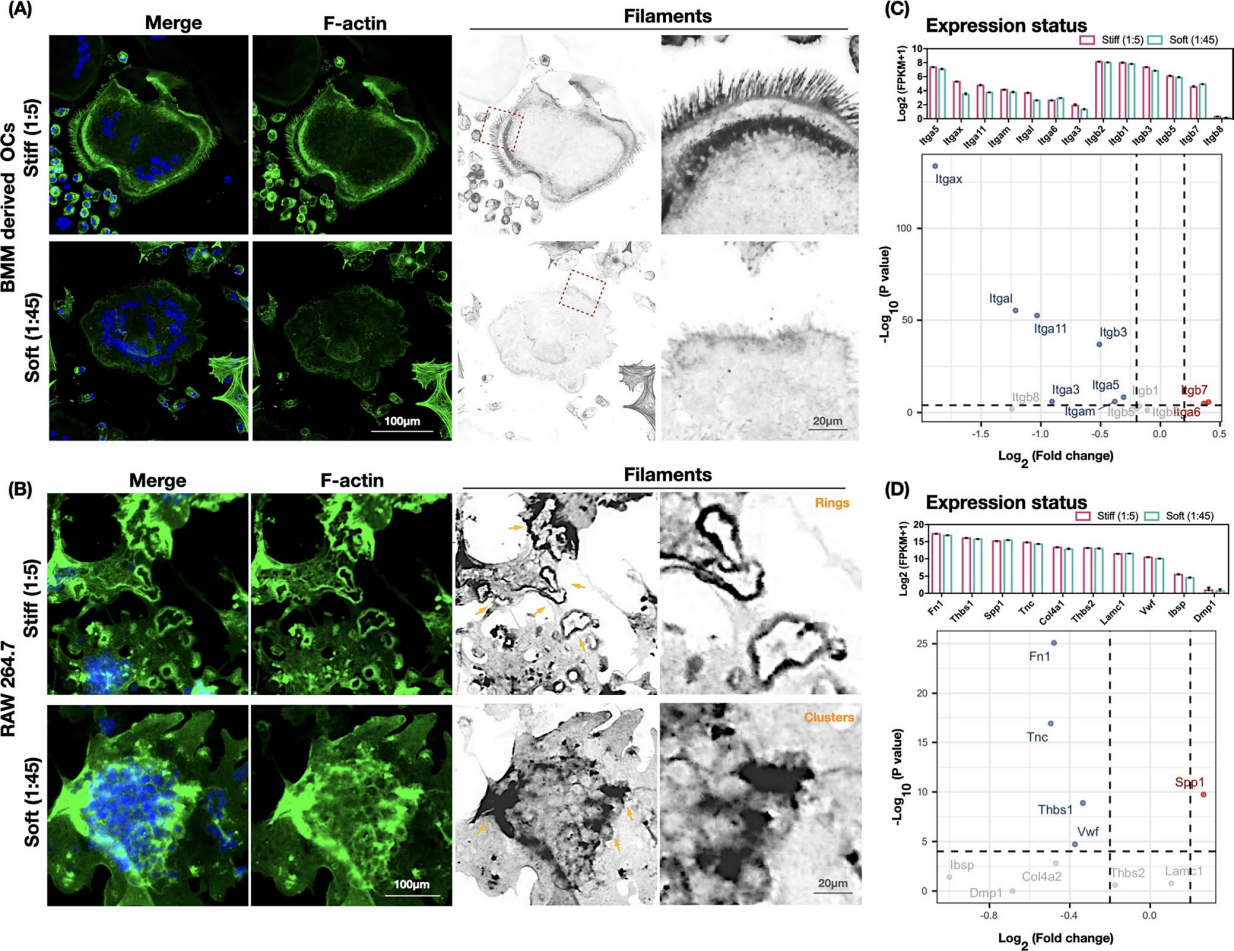
Integrin–ECM profile enrichment of osteoclasts changes with substrate stiffness. (A, B) Remodeling of the cytoskeleton architecture of osteoclasts differentiated from bone marrow precursor cells and RAW 264.7 monocytic cells. Representative immunofluorescence images of osteoclasts counterstained for F‐actin (phalloidin, green) and nuclei (DAPI, blue) display changes in the F‐actin bundles on PDMS substrates of different rigidity (*n* = 3 images). (C) RNA sequencing results indicating the expression landscape of 13 significant genes in the integrin family, screened at padj ≤0.05, in cell samples (*n* = 3 biological replicates) collected from different PDMS substrates (1:5 and 1:45). The upper statistical plot demonstrates the transcriptome expression status of the screened integrin genes, presented as log2 (1 + FPKM). The volcano plots summarize the expression levels of the genes. Comparing soft with stiff samples, downregulated genes are shown as blue dots, upregulated genes as red dots, and nonsignificant genes as gray dots (Threshold: *p* < .0001). (D) RNA sequencing results indicating the expression landscape of integrin αvβ3 interacting with ECM ligands in the ECM–receptor interaction pathways (KEGG) in samples (*n* = 3 biological replicates) collected from PDMS substrates of different rigidity (1:5 and 1:45). The upper statistical plot indicates the transcriptome expression status of select ECM ligands, presented as log2 (1 + FPKM). The volcano plots summarize the expression levels of the ECM ligands. Comparing soft with stiff samples, downregulated genes are shown as blue dots, upregulated genes as red dots, and nonsignificant genes as gray dots (Threshold: *p* < .0001)

The increase in cell size and acquisition of resorption function during osteoclast maturation are accomplished by reconstruction of the adhesive cytoskeleton.^27^, ^28^, ^29^ Thus, the organization of actin filaments in osteoclasts cultured on the PDMS substrates was examined by CLSM. The cytoskeleton architecture of the osteoclasts was notably different on the various substrates. In BMM‐derived osteoclasts on the stiff substrates, the actin filaments were bundled firmly on the cell membrane with multiple cell cilia, whereas in those on the soft substrates, they were less accumulated along the cell border and appeared scrambled (Figure 5A). In RAW 264.7‐differentiated osteoclasts, multiple organized podosome rings were observed on the stiffer substrates, whereas only dotted podosome clusters were seen on the softer substrates (Figure 5B, yellow arrows). These analyses confirmed a significant shift in cytoskeletal (F‐actin) organization in close association with mechanical clues.

To further explore the potential molecules involved in mechanosensing, we focused on the integrin family, which is the prerequisite for cell adhesion and links the ECM to the intercellular cytoskeleton. In total, 13 significantly DEGs from the integrin family were identified between osteoclasts cultured on stiff (1:5) and soft (1:45) substrates. The expression level of these genes (Figure 5C, upper statistical plot) and an overview of their expression differences and statistical level of significance (Figure 5C, volcano plot) were examined. *Itga5* was the most highly expressed among the α‐subunit members. *Itgb3* ranked top three of the β subunits, and although it was expressed at a slightly lower level than *Itgb1* and *Itgb2*, its fold change was clearly higher. Thus, we focused on integrin αvβ3; the expression changes in response to substrate stiffness and of which high enrichment in osteoclasts have previously been reported.^30^ Considering that interactions between integrin αvβ3 and environmental clues are bridged by the ECM,^31^ we compared the expression landscape of integrin αvβ3 interacted ECM ligands between cells on the soft and stiff substrates. The receptor ligands FN1, von Willebrand factor, tenascin C, laminin, integrin‐binding sialoprotein, thrombospondin‐1, thrombospondin‐2, secreted phosphoprotein 1, sialoprotein, dentin matrix protein 1, and collagen type IV were selected from the ECM–receptor interaction pathways (KEGG). Of these, FN1 was the most abundant and also displayed the largest fold change with the highest statistically significant level (Figure 5D).

### 3.6 Substrate stiffness regulated fibronectin–integrin αvβ3 signaling and promoted the expression of downstream intercellular activators

**FIGURE 6:**
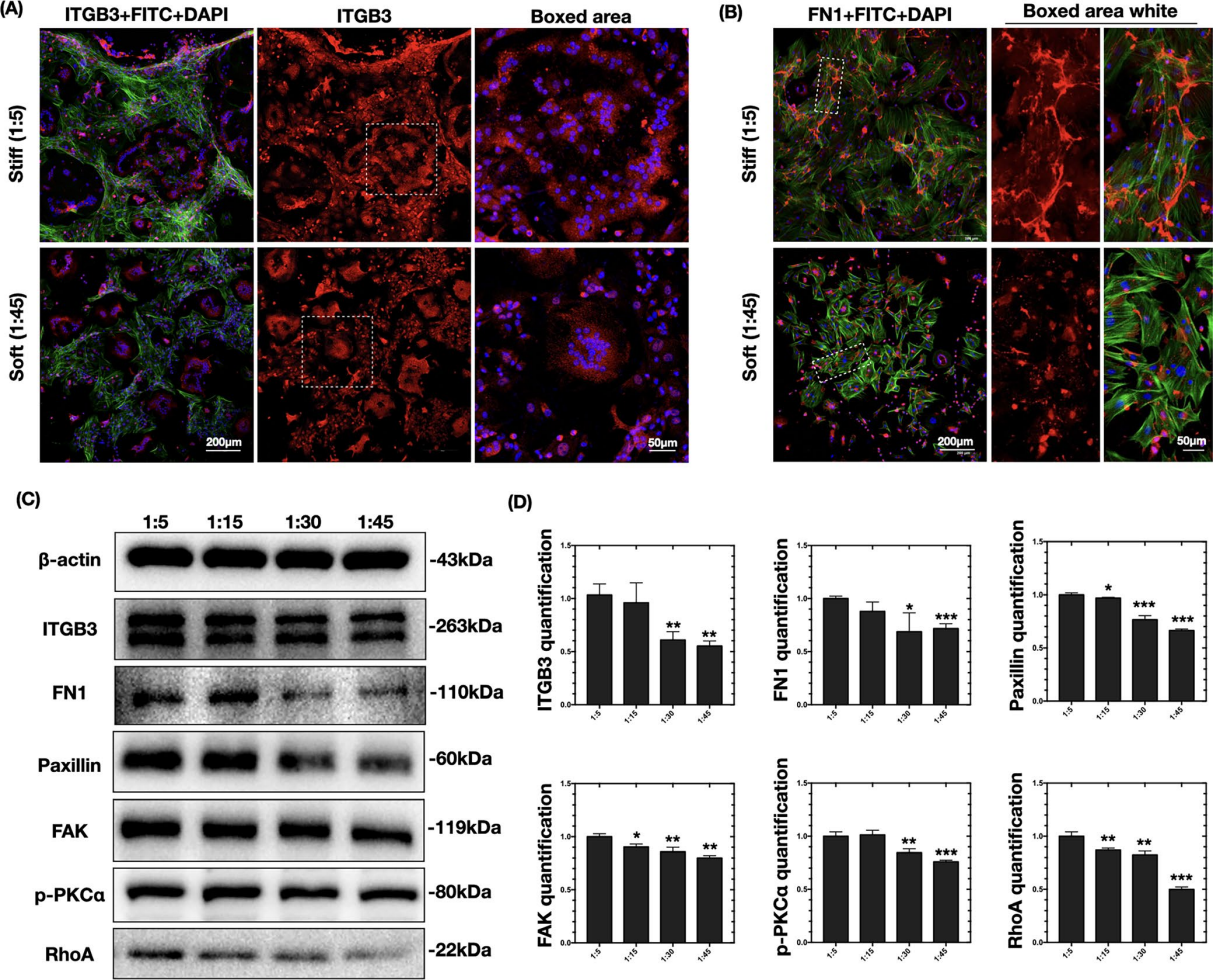
Changes in fibronectin–integrin signaling pathways. (A, B) Representative immunofluorescence images of ITGB3 and FN1 (red stained) on PDMS substrates of different rigidity (*n* = 3 independent experiments). The osteoclasts were counterstained for F‐actin (phalloidin, green) and nuclei (DAPI, blue). (C) Western blots showing the expression levels of ITGB3, FN1, paxillin, FAK, p‐PKC, and RhoA on PDMS substrates of different rigidity (*n* = 3 independent experiments). (D) Quantification of the changes in ITGB3, FN1, paxillin, p‐PKC, FAK, and RhoA levels in (C). Data are the mean ±SD (*n* = 3). *Significant difference relative to group (1:5). *p < .05, **p < .01, **p < .001

Immunofluorescence was applied to detect changes in the distribution of integrin αvβ3 and fibronectin screened in Figure 5C and D. Integrin αvβ3 showed much brighter intensity on the cell border on the stiffer substrates (Figure 6A). Concordant with integrin αvβ3, the branch‐structured fibronectin displayed a high level of continuous deposition along with the cytoskeleton (F‐actin) in the stiffer group, whereas it appeared as fragmented filaments in the softer group (Figure 6B). The variations in integrin αvβ3 and fibronectin protein levels were confirmed by western blot assay and quantified to be significantly reduced on the softer substrates (Figure 6C, D). Therefore, the levels of integrin αvβ3 and fibronectin were verified to be significantly altered in response to substrate stiffness, suggesting they are possible mechanosensors that link microenvironmental clues to the intercellular cytoskeleton.

To confirm the shift in integrin activation, downstream intercellular activators were examined. Paxillin, an important integrin‐associated protein that amplifies the signal of integrin‐induced adhesion,^32^ was confirmed by western blotting to be more highly expressed in cells on stiffer substrates (Figure 6C). FAK,^33^ another integrin‐associated kinase that reinforces the activation of paxillin, was also more highly expressed on stiff substrates. Integrin–adaptor protein intercellular activation is followed by the phosphorylation of PKCα for further reorganization of the actin cytoskeleton.^34^ Moreover, integrin‐mediated PKC activation regulates adhesion and podosome formation in osteoclasts through a RhoA‐dependent pathway.^35^, ^36^ Rho proteins contribute to the reorganization of the actin cytoskeleton and regulate the cell shape.^37^ Similarly, western blotting showed the reduced expression of p‐PKC and RhoA in cells on the soft substrate relative to that on the stiff substrate (Figure 6C, D). Therefore, cytoskeletal organization in response to substrate stiffness in osteoclasts is possibly regulated by fibronectin–integrin αvβ3 signaling pathways.

### 3.7 Prediction of the network of fibronectin–integrin and cytoskeletal signaling molecules and osteoclast differentiation markers altered by substrate stiffness

**FIGURE 7:**
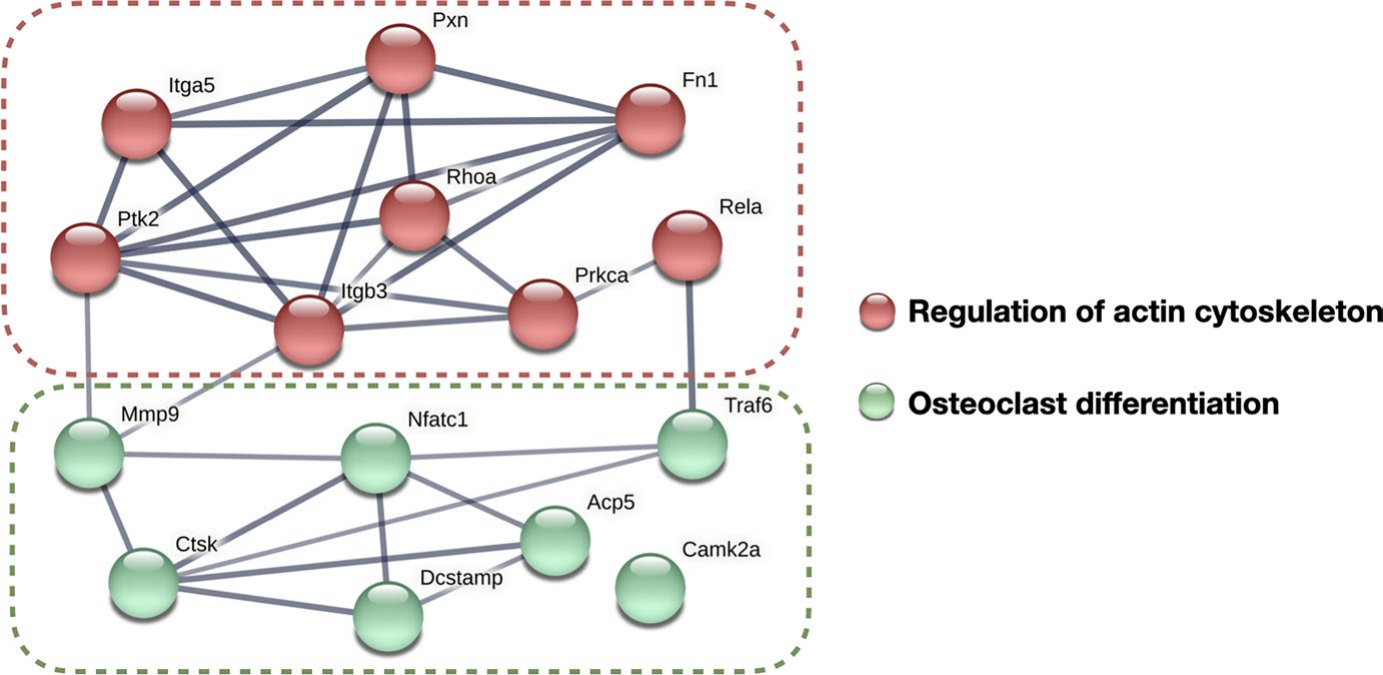
Predicted protein–protein interaction network of fibronectin–integrin signaling molecules and osteoclast differentiation markers. Genes encoding fibronectin–integrin signaling molecules include *Itgb3*, *Itba5*, *Fn1*, *Pxn*, *Ptk2*, *Prkca*, and *RhoA*, as verified by this study. Osteoclast differentiation markers include the downregulated profile (*Nfatc1*, *Ctsk*, *DC*‐*stamp*, *Rela*, *Camk2a*, *Traf6*, *Mmp9*, and *Acp5*) verified in Figure 3. The connection edges shown are of high confidence (0.700), with the thicker connection line indicating the highest confidence (0.900). The network proteins were clustered into two groups using the k‐means method

To investigate the mechanism by which substrate stiffness alters integrin signaling pathways and osteoclast differentiation, we predicted the protein–protein interaction network of fibronectin–integrin and cytoskeletal signaling molecules and osteoclast differentiation markers (Figure 7). The integrin–ECM signaling elements confirmed the integrin αvβ3, FN1, and downstream intercellular activators screened out in this study (Figures 5 and 6). Osteoclast differentiation markers included those that were downregulated (verified in Figure 3). The interacting proteins were clustered into two groups (regulation of actin cytoskeleton and osteoclast differentiation), with a connection confidence higher than 0.7.^22^ Critical nodes connecting osteoclast differentiation to regulation of the actin cytoskeleton were noted. *Itgb3* and *Ptk2* were linked to the majority of actin cytoskeleton elements and were directly connected to *Mmp9*, which contributes to osteoclastic bone resorption. *Prkca* was indirectly linked to *Nfatc1* via *Rela* and *Traf6*. Therefore, substrate stiffness‐regulated integrin–cytoskeleton signaling was predicted to be closely related to osteoclastogenesis markers whose levels were altered under the different stiffness conditions.

## 4 DISCUSSION

The microenvironment of cells provides mechanical signals that ultimately translate into biochemical pathways for cell differentiation and functions.^9^ One important mechanical signal of bone cell surroundings is the bone matrix stiffness. The stiffness of bone tissue can be altered by aging and common diseases that influence the architecture of mineral components (as occurs in osteoporosis, osteogenesis imperfecta, osteoarthritis, and Paget's disease), which changes the microenvironment of the cells.^8^, ^38^ In this study, we established a mechanical model to investigate how osteoclasts sense and react to matrix stiffness. PDMS substrates were selected for their good biocompatibility and wide range of mechanical properties that mimic physiological conditions highly.^39^ Generally, the elasticity of tissues varies widely, ranging in Young's moduli from 0.1 kPa (the softest) in nervous tissue, 10–100 kPa in muscle, around 100 kPa in collagenous bone, and to 15 MPa (the most rigid) in the mineralized bone cortex.^9^, ^40^ By comparison, polyacrylamide gels, which are widely used in studies of cell–substrate interactions, have a narrow elasticity range of 10–100 kPa, making then unsuitable for mimicking bone tissues.^24^ In this study, PDMS substrates with mechanical properties ranging from 0.03 to 4.05 MPa were applied as stiffness signals. The biomaterial topography, especially its roughness gradient, can affect cell behavior.^23^ Because the different substrates showed similarly polished surfaces (defined by *R*a < 0.2 μm),^41^ any influence of the surface topology on osteoclastic responses could be excluded.

During osteoclast differentiation, precursor cells approach and interact with one another to form large polykaryons through several rounds of fusion and fission events.^42^, ^43^ Osteoclasts cultured on the stiffer PDMS substrates displayed a very flexible migration rate, transforming dynamically into large osteoclasts by rapid fusion with surrounding cells within 1–2 h. By contrast, the fusion of small osteoclasts was disabled on the softer substrates. Many molecular factors have been proposed to program fusion events in osteoclasts, with DC‐STAMP considered as the master regulator.^44^ In DC‐STAMP knockout mice, which exhibited osteopetrosis, a mass of small mononuclear osteoclasts with abrogated cell fusion capability were observed.^25^ Other fusion candidates include CD47,^45^ osteoclast stimulatory transmembrane protein (OC‐STAMP),^46^ osteoclast‐associated receptor (OSCAR),^47^ dynamin,^30^ E‐cadherin,^48^ and syncytin‐1.^49^ Moreover, both continuous fusion events and self‐recycling by fission alter the fate of short‐lived osteoclasts via transcriptional activity.^50^, ^51^ Thus, we further analyzed the expression of osteoclast differentiation markers under stiffness stimulation. Canonical osteoclast markers (Nfatc1, Acp5, Ctsk, Camk2a, Mmp9, and Traf6) and the fusion master regulator DC‐stamp were highly expressed on the stiffer substrates, indicating the presence of mature osteoclasts, which was further confirmed by analysis of the cell resorption functions and live imaging observations. Taken together, the alterations of osteoclast morphology, fusion/fission activity, and resorption function and osteoclastogenesis profiles confirm that osteoclast differentiation reacts strongly to stiffness signals.

Notably, the architecture of cytoskeletal actin filaments was significantly altered by substrate stiffness stimulation. The rearrangement of cytoskeleton‐associated adhesion molecules upon the mechanosensing of ECM stiffness has been observed in mesenchymal stem cells,^9^ apical papilla‐derived stem cells,^52^ adipose‐derived stromal cells,^53^ and bone cells such as osteoblasts,^10^ osteocytes,^11^ and chondrocytes.^12^ Characterization of the responses of mechanosensitive structures to stiffness signals has implicated integrin‐mediated cell adhesion, cell–ECM interaction, integrin adaptor proteins, and cytoskeletal elements in a coordinated structural network.^32^ During this process, the mechanical force triggers rapid conformational changes in integrin, activating downstream force‐sensitive adhesion proteins (paxillin, talin, FAK, Src, Rho family GTPases, etc.), followed by biochemical signaling cascades of cytoskeletal reorganization mediated by actin polymerization and actomyosin contractility, leading to long‐term changes in the cellular behavior and differentiation profiles.^54^ Of note, the specific actin structures named podosomes displayed distinct patterns on substrates of different stiffness. Podosomes are another class of cell–matrix adhesions mediated by integrins, are detected in cells from the monocytic lineage, and play a key role in the formation of sealing zones in osteoclasts for bone resorption.^55^, ^56^ Recent studies have proposed a new role of podosomes as mechanosensitive structures initiated by integrins that signal similarly to focal adhesions.^56^, ^57^ Integrin αvβ3, which is highly expressed in osteoclasts,^30^ was identified as a potential responder to mechanical signals in this study. The enrichment of integrin αvβ3 interactions with ECM ligands and the activation of intergrin downstream molecules (paxillin, FAK, PKCα, and RhoA) provide evidence that substrate stiffness is a strong signal for osteoclasts, possibly initiating cytoskeletal reorganization through regulation by fibronectin–integrin‐initiated adhesion pathways. Furthermore, multiple connections of high confidence were observed in the predicted protein–protein interaction network of fibronectin–integrin signaling molecules and osteoclast differentiation markers.

In conclusion, we have revealed for the first time that extracellular substrate stiffness is a strong determinant of the morphology, fusion activity, transcription profile, and resorption functions of osteoclasts, with stiffer substrates acting as a signal booster for osteoclast differentiation. Osteoclasts sense these mechanical signals and respond via cytoskeleton‐associated adhesion molecules, including fibronectin–integrin αvβ3 activation, and following biochemical signaling cascades of paxillin, FAK, PKC, and RhoA. The bioinformatics data proposed a strong connection between cytoskeletal adhesion and osteoclast differentiation. The genes in the connection nodes should be confirmed in our next study. This study contributes deeper knowledge about osteoclastogenesis from the view of mechanical stimulation by biomaterials that mimic the in vivo microenvironmental stiffness, which osteoclasts sense from physical to pathological conditions. The results provide evidence of the mechanical regulation of osteoclast activity in bone homeostasis and diseases.

## CONFLICT OF INTEREST

The authors declare no conflict of interest.

## AUTHOR CONTRIBUTIONS

Qingxuan Wang, Wenli Lai, and Chenchen Zhou designed the experiments. Qingxuan Wang and Jing Xie performed the experiments. Qingxuan Wang and Chenchen Zhou analyzed and confirmed all data and edited the manuscript. All authors reviewed and approved the final paper.

## Supporting information

Video S1

Video S2

## Data Availability

The data supporting the results of this study are available upon request from the corresponding author.
